# Supplementary material for: Effect of β-caryophyllene from Cloves Extract on Helicobacter pylori Eradication in Mouse Model
Source: Nutrients. 2020 Apr 4;12(4):1000. doi: 10.3390/nu12041000 (PMC7230661; doi:10.3390/nu12041000)

Supplementary Figure S1. In vitro effect of H-002119-00-001 on bacterial colonization (A) After treatment with 0.1 M H-002119-00-001, the number of bacteria showed over 3.0 x 10^2 CFU/mL. (B) After treatment with 0.01 M H-002119-00-001, the number of bacteria showed over 3.0 x 10^2 CFU/mL. (C) After treatment with 0.001 M H-002119-00-001, the number of bacteria showed over 3.0 x 10^2 CFU/mL.

(A)

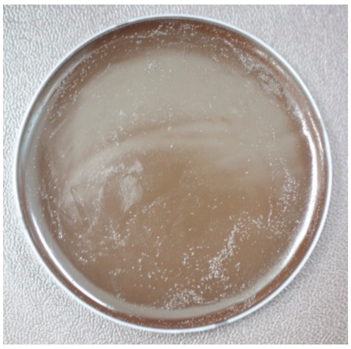

(B)

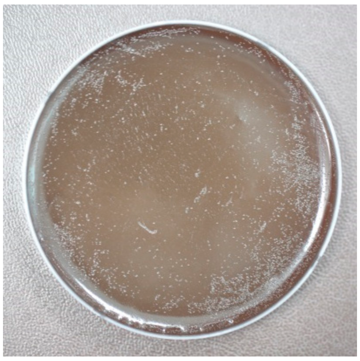

(C)

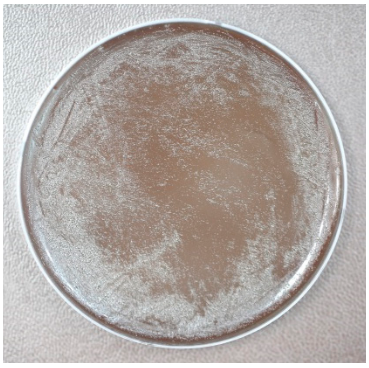

Supplement: Supplementary file 1 [file nutrients-12-01000-s001.pdf]
